# Thermal Degradation Study of Hydrogel Nanocomposites Based on Polyacrylamide and Nanosilica Used for Conformance Control and Water Shutoff

**DOI:** 10.3390/gels10120846

**Published:** 2024-12-22

**Authors:** Aleksey Telin, Farit Safarov, Ravil Yakubov, Ekaterina Gusarova, Artem Pavlik, Lyubov Lenchenkova, Vladimir Dokichev

**Affiliations:** 1 Ufa Scientific and Technical Center, LLC., 450078 Ufa, Russia; safarovfi@ufntc.ru; 2 Ufa Institute of Chemistry, Ufa Federal Research Center, Russian Academy of Sciences, 450054 Ufa, Russia; 3 Faculty of Mining and Petroleum, Ufa State Petroleum Technological University, 450064 Ufa, Russialnplay@mail.ru (A.P.);; 4 Interdisciplinary Research Laboratory of Oilfield Chemistry, Ufa University of Science and Technology, 450008 Ufa, Russia

**Keywords:** water shutoff, conformance control, hydrogels, nanoparticles, thermodestruction, rheological studies, filtration studies

## Abstract

The application of nanocomposites based on polyacrylamide hydrogels as well as silica nanoparticles in various tasks related to the petroleum industry has been rapidly developing in the last 10–15 years. Analysis of the literature has shown that the introduction of nanoparticles into hydrogels significantly increases their structural and mechanical characteristics and improves their thermal stability. Nanocomposites based on hydrogels are used in different technological processes of oil production: for conformance control, water shutoff in production wells, and well killing with loss circulation control. In all these processes, hydrogels crosslinked with different crosslinkers are used, with the addition of different amounts of nanoparticles. The highest nanoparticle content, from 5 to 9 wt%, was observed in hydrogels for well killing. This is explained by the fact that the volumes of injection of block packs are counted only in tens of cubic meters, and for the sake of trouble-free workover, it is very important to preserve the structural and mechanical properties of block packs during the entire repair of the well. For water shutoff, the volumes of nanocomposite injection, depending on the well design, are from 50 to 150 m^3^. For conformance control, it is required to inject from one to several thousand cubic meters of hydrogel with nanoparticles. Naturally, for such operations, service companies try to select compositions with the minimum required nanoparticle content, which would ensure injection efficiency but at the same time would not lose economic attractiveness. The aim of the present work is to develop formulations of nanocomposites with increased structural and mechanical characteristics based on hydrogels made of partially hydrolyzed polyacrylamide crosslinked with resorcinol and paraform, with the addition of commercially available nanosilica, as well as to study their thermal degradation, which is necessary to predict the lifetime of gel shields in reservoir conditions. Hydrogels with additives of pyrogenic (HCSIL200, HCSIL300, RX380) and hydrated (white carbon black grades: ‘BS-50’, ‘BS-120 NU’, ‘BS-120 U’) nanosilica have been studied. The best samples in terms of their structural and mechanical properties have been established: nanocomposites with HCSIL200, HCSIL300, and BS-120 NU. The addition of hydrophilic nanosilica HCSIL200 in the amount of 0.4 wt% to a hydrogel consisting of partially hydrolyzed polyacrylamide (1%), resorcinol (0.04%), and paraform (0.09%) increased its elastic modulus by almost two times and its USS by almost three times. The thermal degradation of hydrogels was studied at 140 °C, and the experimental time was converted to the exposure time at 80 °C using Van’t Hoff’s rule. It was found that the nanocomposite with HCSIL200 retains its properties at a satisfactory level for 19 months. Filtration studies on water-saturated fractured reservoir models showed that the residual resistance factor and selectivity of the effect of nanocomposites with HCSIL200 on fractures are very high (226.4 and 91.6 for fracture with an opening of 0.05 cm and 11.0 for porous medium with a permeability of 332.3 mD). The selectivity of the isolating action on fractured intervals of the porous formation was noted.

## 1. Introduction

The application of polyacrylamide hydrogels crosslinked with Cr^3^⁺ or Al^3^⁺ ions for conformance control and water shutoff has become a well-established practice in the oil industry, as it is a successful approach to mitigating the problem of early water breakthrough in waterflooded fields [1,2].

**Figure 1:**
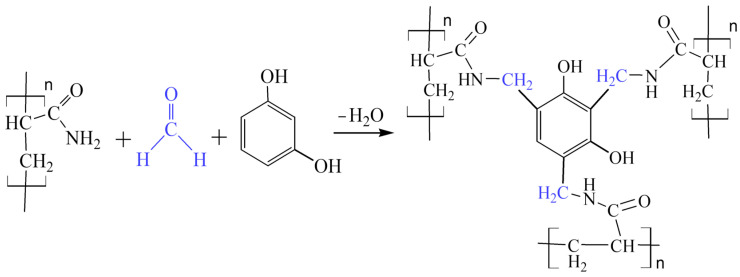
Schematic of the crosslinking reaction of polyacrylamide with paraform and resorcinol.

The crosslinking of partially hydrolyzed polyacrylamide with resorcinol and paraform for these purposes has also been carried out for a long time [3,4,5]. Figure 1 shows the crosslinking of the amide fragment of the polymer resulting from the aminomethylation reaction.

The use of organic crosslinkers of polyacrylamide is preferable to the use of Cr^3^⁺ or Al^3^⁺ ions because the covalent bonds in the hydrogel mesh structure provide better rheological characteristics and allow a smoother adjustment of properties depending on the concentrations of the components [6,7].

It should be noted that hydrogels based on partially hydrolyzed polyacrylamide have been increasingly modified with nanoparticles in recent years. The fact is that, under harsh conditions of temperature and salt aggression, the decomposition of gel shields based on crosslinked polymer systems occurs. The degradation of hydrogels consists of the rupture of the main polymer chains, crosslinking rupture, polymer hydrolysis, and syneresis [8]. The silanol groups of nanosilica particles interact with the carboxyl groups of partially hydrolyzed polyacrylamide to form a hydrogen bond (proved by IR spectra) [9], thus enhancing the interaction with water molecules and preventing the dehydration and syneresis of the gel [10]. At the same time, nanoparticles are uniformly distributed throughout the hydrogel volume. In [11], it is shown that the introduction of 9% nanosilica increases the strength of hydrogel by 5000%. Electron microscope images show that the nanoparticles reinforce the hydrogel during in situ gelation, which allows the nanocomposite to resist temperature, salt, and shear stresses much better then hydrogel without the addition of nanosilica. Article [12] provides data that the addition of 8% of nanosilica to the gel based on partially hydrolyzed polyacrylamide and the environmentally friendly crosslinker polyethylenimine provides an increase in the modulus of elasticity by 14 times. For high-temperature reservoir applications, nanocomposite gel is obtained from thermally stable sulfonated polyacrylamide with the addition of nanoparticles—secondary-modified laponite, thiourea, and crosslinker polyethylenimine [13]. The authors of article [14] noted that the linear dimensions of nanosilica significantly affect the properties of nanocomposites. Thus, hydrogel based on partially hydrolyzed sulfonated polyacrylamide crosslinked with chromium acetate has the maximum strength characteristics when combined with nanoparticles with a size of 20–30 nm compared to similar gels with the addition of nanosilica with sizes of 7–10 nm and 60–70 nm. The influence of the linear size of nanoparticles on the properties of nanocomposites was also noted in [15]. The same authors, in [16], synthesized porous polyacrylamide hydrogels with improved mechanical properties and uniform pore distribution by leaching chemically modified silica particles from the hydrogel. Such an approach in the future should allow obtaining organic–inorganic hydrogels with a complex of unique properties. At the same time, the amount of nanoparticles that is introduced should provide better performance of the hydrogel while remaining commercially attractive.

Most authors emphasize that the introduction of nanosilica into hydrogels increases their viscoelastic and viscoplastic properties, as well as increasing their thermal stability due to the formation of multiple hydrogen bonds [17,18]. Both hydrophilic and hydrophobic nanosilica of pyrogenic origin [19] as well as hydrated nanosilica [20] have been used for this purpose.

The introduction of nanosilica also increases the resistance of hydrogel to thermal degradation due to the formation of new nodes in the crosslinked polymer network [21]. It should be noted that the study of nanocomposites based on polyacrylamide hydrogels has been rapidly developing over the last 10–15 years and is one of the most promising applications of gels in the oil industry: for water shutoff, conformance control, and well killing with loss control [22,23,24]. All these listed applications of nanoparticle-enhanced hydrogels have significantly expanded the boundaries of their applicability under conditions of thermal and salt aggression. Nevertheless, the question of the time interval of the thermal stability of hydrogels is especially important from the point of view of predicting the duration of their effect in their application in high-temperature formations and has not yet found sufficient coverage in the literature. The articles on the thermodegradation of hydrogels are mainly devoted to the study of the mechanism of the process [8]. The kinetics of temperature decomposition of hydrogels were studied by a number of authors in relation to the issues of well killing with real-time loss circulation control. The most detailed study of this kind was carried out in [25], where a mathematical model was developed based on the results of experiments with self-destructing hydrogel, which coincides with the measured values of viscosity during the hydrogel’s temperature aging with a correlation coefficient of 0.988. This approach allows predicting the time period for which block compounds will remain in a serviceable condition and estimating the safe period of workover operations at wells, which is calculated to be from 2–3 to 10–15 days. The lifetime of hydrogel screens during conformance control or well water shutoff operations should be at least a year, and we have not found any information on their accelerated temperature aging. In addition, the present work, along with its scientific component, has a clear practical orientation. It considers two important practical aspects: obtaining nanocomposites with good structural and mechanical properties that have a minimum nanoparticle content, ensuring the efficiency of their injection, and obtaining scientifically justified nanocomposite lifetimes in reservoir conditions, which is necessary for predicting the economic efficiency of the technological operation.

Earlier, we studied the effect of thermal degradation on the properties of hydrogels based on sulfonated polyacrylamide samples and came to a conclusion about the technologically acceptable lifetime of gels in which their rheological characteristics in the oil reservoir are preserved [26,27]. It should also be emphasized that previous studies on the thermostability of hydrogels were conducted in sealed glass ampoules. In field practice, hydrogels are in contact with steel surfaces during repair works for the elimination of casing leaks. Considering this, the present studies were carried out in a steel autoclave in order to model the influence of reservoir temperature conditions on the operational properties of the composition during a long period after its injection. The essence of the experiment was to test the stability of the composition with respect to its thermal degradation at 140 °C. The choice of the accelerated thermodestruction temperature in our previous studies allowed us to predict a long (one year and more) lifetime of gel shields based on sulfonated polymers, which coincides with the estimations made in the monograph [28], as well as with the results of laboratory studies and field operations with sulfonated polymers crosslinked with chromium acetate [29]. Thus, accelerated thermal degradation of the nanocomposite takes place, which makes it possible to simulate the effect of a reservoir temperature of 80 °C for a large time interval, while the experiment itself takes much less time. For the artificially accelerated aging of hydrogels, we used Van’t Hoff’s rule that the rate of chemical reaction increases by two to four times for every 10 °C increase in temperature. The results of the artificial aging of the nanocomposites (rheological characteristics) obtained at 140 °C were extrapolated for a time that corresponds to a reservoir temperature equal to 80 °C.

The aim of this work is to develop formulations of nanocomposites based on polyacrylamide hydrogels crosslinked with resorcinol and paraform, with the addition of commercially available nanosilica, that have enhanced structural and mechanical characteristics, and to study their thermal degradation, which is necessary to predict the lifetime of gel shields consisting of nanocomposites in reservoir conditions.

The novelty of this study lies in the systematic study of the influence of different origin nanosilica and wettability surface properties on the structural and mechanical characteristics of nanocomposites and the time of preservation of their properties in reservoir conditions.

## 2. Results and Discussion

Taking into account the information available in the literature [19], which indicates that both hydrophilic and hydrophobic nanoparticles increase the structural-mechanical properties and thermal stability of hydrogels, we conducted a preliminary series of experiments by introducing additives of hydrophilic (HCSIL200) and hydrophobic (RX380) nanosilica into the hydrogel. In all experiments, the nanosilica was added in concentrations from 0.1 to 0.5% in increments of 0.1%. The measured parameter was the value of ultimate shear stress (USS), Pa, which is easily determined in shear tests. It was found that the basic value of the USS of the hydrogel without additives (base gel) was equal to 104 Pa. The results of the remaining experiments are shown in Table 1. It can be seen in the table that the addition of hydrophilic nanosilica in the amount of 0.5% increases the USS by more than 3 times, and the addition of hydrophobic nanosilica increases the USS by less than 1.5 times.

It was decided to carry out further oscillation experiments with a 0.4% nanosilica additive since the USS values obtained by increasing the additive content up to 0.5% were insignificantly different. The value of the ultimate shear stress obtained with the addition of 0.4 wt% hydrophilic nanosilica, equal to 290 Pa, is quite acceptable for the regulation of water flows in the reservoir during well water shutoff and conformance control operations [30]. Furthermore, economic considerations were also taken into account.

It should be noted that an increase in the content of hydrophobic nanosilica reduces the gelation time, while the addition of hydrophilic nanosilica does not affect this parameter, and all the obtained gelation times are quite comfortable for working on wells. The variation in gelation time in the presence of silica nanoparticles with different wettabilities seems to be related to the process of diffusion inhibition of the crosslinking reaction of partially hydrolyzed polyacrylamide. The formation of the grid in this case occurs due to the polycondensation reaction of resorcinol and paraform with the amide group of the polymer. In aqueous media, the behavior of hydrophobic particles is determined by hydrophobic interactions due to van der Waals forces between the main chain of the polymer and the hydrophobic nanosilica. In this case, the functional amide group is shielded to a lesser extent than in the case of the hydrophilic nanosilica, which interacts with the functional groups of the polymer due to the formation of hydrogen bonds.

The next series of experiments was carried out with a number of samples of commercially available nanosilica, both of pyrophoric origin and precipitated from dispersions of sols, the so-called white carbon black samples, which differ from pyrophoric ones by a small degree of hydration. Oscillation tests were carried out with determination of the elastic modulus (Pa), viscous modulus (Pa), complex modulus (Pa), complex viscosity (Pa·s), crossover point (Pa), and linear viscoelastic region (LVR, Pa). The experimental results (mean values and standard deviations of the determined parameters) are given in Table 2.

From the data in Table 2, it can be seen that the greatest increase in properties is observed with the addition of hydrophilic pyrogenic silica (HCSIL200, HCSIL300)—the elastic moduli increased from 36.1 Pa to 57.3 and 49.7, respectively. The value of the crossover point, which can be interpreted as the yield strength of the hydrogel, increased markedly. Thus, for the base gel it was 50.5 Pa, and when HCSIL200 and HCSIL300 nanoparticles were added, it increased to 67.4 and 82.9 Pa, respectively. The application of hydrophobic pyrogenic nanosilica (RX380) and hydrated silica (white carbon black of BS-50 grade) did not lead to significant changes in the properties of the studied composition in both cases. However, when the particle sizes of the white carbon black are reduced (BS-120 NU and BS-120 U grades), there is an increase in the considered structural and mechanical characteristics. Thus, the best results for the compositions with hydrated silica were shown by nanomaterial BS-120 NU; namely, the elastic modulus was 45.7 Pa, the value of the crossover point was 59.6 Pa, and the linear viscoelastic region was 34.5 Pa.

The results of the oscillation studies allowed us to identify the most effective nanodisperse filler—hydrophilic pyrogenic silicon dioxide of HCSIL200 grade. It significantly increases both the complex modulus of elasticity and the yield strength of the hydrogel. However, the results of the oscillation studies of the nanocomposite with BS-120 NU are not so significantly different from the results for the compositions with hydrophilic pyrogenic silica. Taking into account the economic availability of this material, it can be concluded that the application of white carbon black BS-120 NU as a nanodispersed additive in hydrogels to improve their structural and mechanical properties is promising.

The structure and surface properties of white carbon black, hydrophobic silica RX380, and hydrophilic nanosilica HCSIL200 and HCSIL300 differ significantly. The hydrophilic amorphous nanosilica HCSIL200 and HCSIL300, as well as white carbon black, are characterized by the presence of silanol groups on the surfaces of the particles, capable of forming hydrogen bonds with carboxyl and amide functional groups of the crosslinked polymer. Hydrophobic silica RX380 is a chemically modified amorphous silica obtained by surface activation followed by modification with organosilane precursors, which suppresses water condensation on the silica surface. This sample is not characterized by the formation of hydrogen bonds, and the interaction with the crosslinked polymer occurs in aqueous medium due to van der Waals forces.

Having selected the best nanocomposite sample (HCSIL200) on the basis of rheological testing, we conducted filtration testing with it on a water-saturated ideal fracture and composite models of porous medium made of natural core material under reservoir thermobaric conditions. The aim of the experiment was to simulate conformance control in carbonate reservoirs with double permeability.

**Figure 2:**
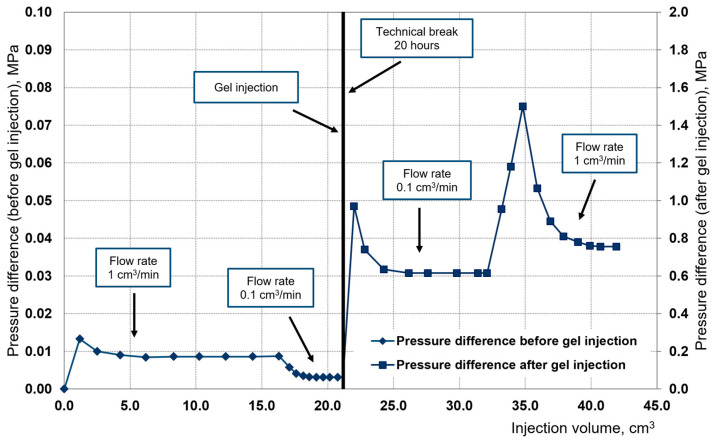
Dynamics of pressure drop variation from injection volume.

The dynamics of changes in the main indicators during the filtration experiment on the slot model are presented in Figure 2.

When the flow rate was increased by an order of magnitude from 0.1 cm^3^/min to 1.0 cm^3^/min, there was no erosion of the hydrogel, which can be seen from the increase in the critical pressure gradient and stabilization pressure gradient. The residual resistance factor at a flow rate of 0.1 cm^3^/min was 226.37, and at a flow rate of 1 cm^3^/min, it was 91.55.

**Figure 3:**
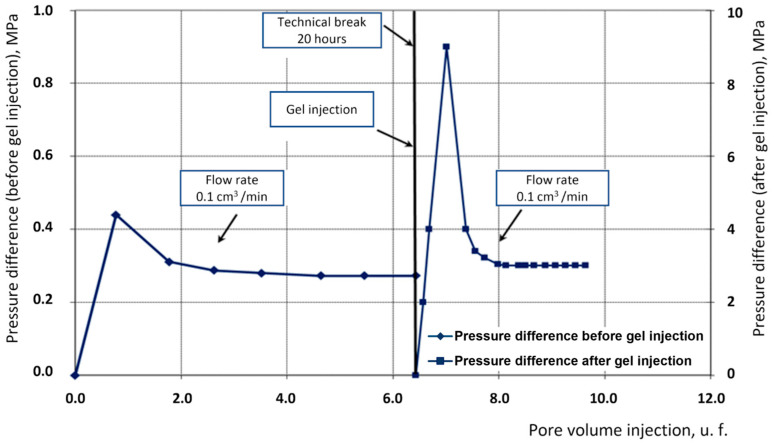
Dynamics of pressure drop variation from pore volume injection.

The second experiment was conducted on a composite porous medium. The residual resistance factor in this experiment was 11.05 (Figure 3).

The main results of the filtration experiments are presented in Table 3.

The results of these two experiments show that the developed composition exhibits selectivity in permeability, which, in turn, is especially valuable for reservoirs with double permeability. According to data in the literature [2], such behavior is characteristic only for crosslinked polymer compositions.

In the course of physicochemical, rheological, and filtration studies, it was found that the composition based on partially hydrolyzed PAM crosslinked by complex organic crosslinker with the addition of nanoparticles has exceptional structural and mechanical properties, exceptional selectivity for permeability, and an exceptional pore space structure. In order to confidently recommend this composition for field tests, it is necessary for it to withstand reservoir temperatures for at least one year. For this purpose, we studied the thermal destruction at 140 °C of the two best compounds in terms of their rheological characteristics: that with the addition of 0.4% of pyrogenic (HCSIL200) and that with the addition of hydrated (BS-120 NU) nanosilica, respectively. The determinable parameter was the USS of the samples subjected to thermal degradation. The experimental results and photos of nanocomposite samples after thermal exposure are presented in Table 4.

As can be seen from Table 4, the nanocomposite with the HCSIL200 additive is the most thermally stable composition, retaining its properties for 19 months. As for the nanocomposite with white carbon black, it is significantly inferior to the leader, and after the specified time, it has approximately the same performance as the hydrogel without nanoparticle additives. Rheological parameters were determined for the degraded nanocomposite samples by oscillatory rheometry, which showed a multiple-fold decrease in performance, as in the shear tests. In particular, the elastic modulus of the HCSIL200 nanocomposite decreased from 57.3 Pa to 33.5 and 13.7 Pa in 9 and 19 months, respectively (time converted to 80 °C according to Van’t Hoff’s equation), while for the 0.4% BS-120 NU nanocomposite, the elastic modulus decreased from 45.7 Pa to 12.7 Pa in 19 months. It should be noted that the indicators of the nanocomposites after thermal degradation for 19 months can be considered as the lower limit of their presence in reservoir conditions in a serviceable state.

This study concludes that hydrophilic nanosilica enhances the structural and mechanical properties of nanocomposites and prolongs their preservation in reservoir conditions. This fact is true for nanocomposites based on partially hydrolyzed polyacrylamide crosslinked with resorcinol and paraform. If other crosslinkers, such as polyethylenimine, chromium, or aluminum salts, are used, the optimal compositions of nanocomposites may change.

## 3. Conclusions

The influence of the addition of a number of commercially available nanosilica samples as structuring additives on the structural and mechanical properties of hydrogel based on partially hydrolyzed polyacrylamide crosslinked with resorcinol and paraform has been studied. As a result of our systematic study of the different origin nanocomposites’ properties and the wettability surface properties of the nanoparticles, the most optimal options for their use in conformance control and water shutoff technologies are determined. It was found that all studied samples of pyrogenic, including hydrophilic and hydrophobic, as well as hydrated nanosilica, increase in different degrees the elastic modulus and complex viscosity of the hydrogel. The hydrophilic nanosilica HCSIL200 has the greatest effect on the hydrogel properties; with the addition of only 0.4% wt., the elastic modulus of the nanocomposite increases from 36.1 to 57.3 Pa.

The study of the accelerated thermodegradation of this nanocomposite allowed us to estimate the lifetime of the insulating gel shield in reservoir conditions at 80 °C. It was found that, at reservoir temperature, the properties of the hydrogel nanocomposite are maintained at an acceptable level for 19 months. In particular, the ultimate shear stress was 116 Pa, and the elastic modulus was 13.7 Pa.

Considering that most large and medium-sized fields developed by the waterflooding method have a high water cut, the noted improvement in conformance control and water shutoff technologies using nanocomposites will help to prolong the profitable operation of oil fields at the last stage of development.

## 4. Materials and Methods

### 4.1. Hydrogel and Additives

The base hydrogel was prepared from partially hydrolyzed polyacrylamide (1.0%) and a complex crosslinker consisting of paraform (0.09%) and resorcinol (0.04%) [26]. The following materials were used: polyacrylamide EOR-1141 (supplier: “Himintek” Ltd., Perm, Russia) with a molecular weight of 3·10^6^ Da and a degree of hydrolysis of 30%; paraform TU 6-09-141-03-89 with modification 1.2; resorcin technical grade 1 OKP 2472110130 (production of JSC “Uralchimplast”, Nizhny Tagil, Russia).

Dispersed additives were: white carbon black of the following grades: “BS-50”, “BS-120 uncompacted” (“BS-120 NU”), “BS-120 compacted” (“BS-120 U”) (production of JSC “Bashkir Soda Company”, Sterlitamak, Russia); hydrophilic nanosilica of grades HCSIL200 (production of Shandong Haochuang Material Co, Ltd., Dezhou, China), HCSIL300 (produced by Shandong Haochuang Material Co., Ltd., Dezhou, China), hydrophobic modified amorphous silica RX380 TU2458-010-3851 8981-2012 (with revision № 1) (produced by RDC “InTechService”, Ufa, Russia).

The choice of specific types of nanosilica was determined by their commercial availability. All the studied samples, including hydrophilic and hydrophobic silica of pyrogenic origin, as well as hydrated silica obtained by precipitation, are produced in large volumes in industry.

Characteristics of the nanoadditives are given in Table 5. The primary particle size was recalculated by Equation (1) from the specific surface of the additive presented in the technical data sheets (the specific surface of HCSIL200 and HCSIL300 was measured by the manufacturer using the BET (Brunauer-Emmett-Teller) method). BS-50, BS-120 U, BS-120 NU were measured by the phenol adsorption method.

(1) $$d=\frac{6000}{\rho \cdot s}$$

where *d* is the calculated particle size, nm; *ρ*—density of silicon dioxide, taken as 2.2 cm^3^/g; *s*—specific surface of the additive, m^2^/g.

The compositions were prepared in fresh water on a magnetic stirrer. The hydrophilic nanoadditives were stirred for 10–15 min until uniform distribution in the volume was obtained. Preparation of the suspension of hydrophobic nanosilica (RX380) included its preliminary dispersion in 2 mL of isopropyl alcohol (grade “chemically pure”). The resulting suspension was poured into water. Due to this, we achieved a uniform distribution of hydrophobic particles in the aqueous solution. Then, the polymer with crosslinkers was added to the solution and stirred for 45 min. After 48 h (gelation and gel maturation time), rheological studies were performed.

### 4.2. Rheological Studies

Ultimate shear stress was determined on a Haake Viscotester iQ rotational viscometer (Thermo Fisher Scientific, Waltham, MA, USA) by the dependence of shear rate on shear stress. For this purpose, the rheological curve was taken in the controlled shear stress (CS) mode. An aliquot of gel in a volume of 4 mL was placed in a measuring cylinder of the CC16 Din/Ti type, and then this cylinder was placed in the thermostated compartment of the rotational viscometer, the rotor was lowered, and the measurement was started. When the USS was reached, a sudden jump in shear rate occurred, and the instrument stopped the measurement. After the measurement was completed, the results were processed and the corresponding “shear rate–shear stress” relationship was plotted. USS was determined by a sharp inflection on the “shear rate–shear stress” curve.

**Figure 4:**
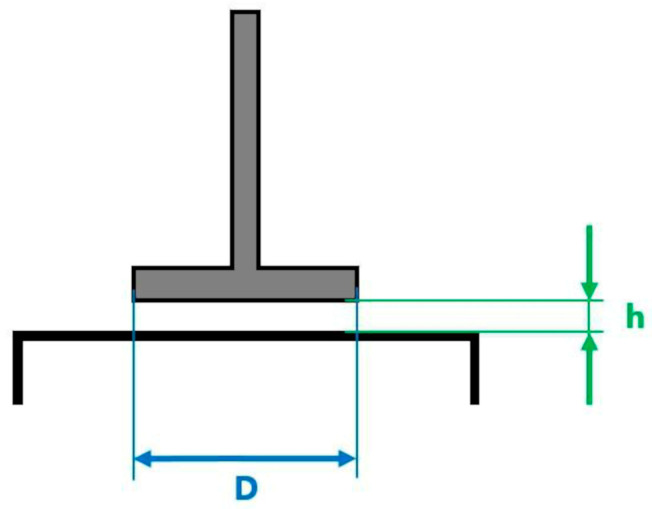
Plate-to-plate measuring system.

Oscillation studies were carried out on a rotational viscometer Rheotest RN 5.1 (RHEOTEST Medingen GmbH, Ottendorf-Okrilla, Germany) with a “plate–plate” measuring system at a temperature of 24 °C. The diameter of the measuring plate D = 36 mm, and the gap between the plates h = 1 mm (Figure 4).

The required volume of hydrogel was applied to the plate using a syringe-doser, and then a gap of 1 mm between the plates was set using a micrometer. The excess hydrogel was removed with special tongs so that the space between the plates was completely filled with the measured gel.

Oscillation studies were performed with a sweep in shear stress τ, at an oscillation frequency ν of 1 Hz. The main parameters measured were the elastic modulus G’, the viscous modulus G’’, the crossover point (point of intersection of G’ and G’’) corresponding to the yield strength, and the LVR. Several measurements were made during the experiments, the results of which were then averaged, and the standard deviation was calculated.

The linear viscoelastic region (LVR) is determined from the measured G’ data. Physically, this corresponds to the onset of significant loss of elasticity with increasing stress.

The modulus of elasticity G’ is the real part of the complex modulus of elasticity; it is tabulated by the rheometer as a function of shear stress values. It shows the elastic properties of the medium.

The viscosity modulus (loss modulus) G’’ is the value of the imaginary part of the complex modulus of elasticity; it is given by the rheometer in tabular form depending on the values of shear stress. This metric displayed losses due to viscous friction.

When the crossover point is reached, the body loses its elastic properties and becomes a fluid medium (liquid).

### 4.3. Thermal Degradation Studies

**Figure 5:**
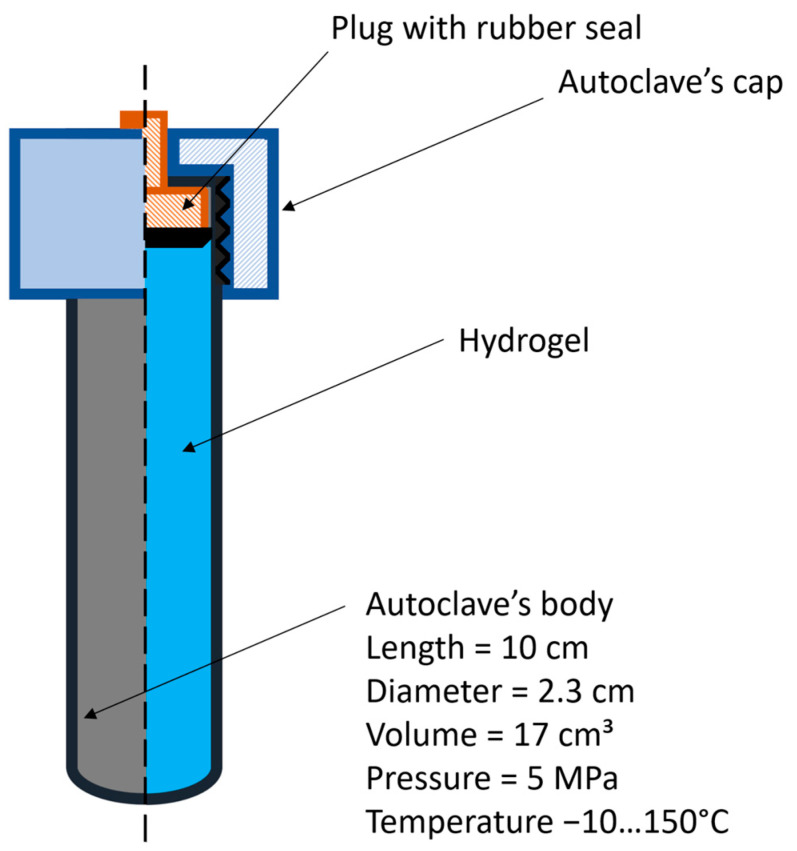
Sketch of finger-type autoclave.

The tests were carried out in finger-type autoclaves (Ufa Institute of Chemistry of RAS, Ufa, Russia) with a volume equal to 17 mL without dynamic loading (Figure 5).

To ensure reproducibility of the thermal degradation experiments, 14 mL of hydrogel was placed in a 17 mL autoclave, and the remaining free volume was purged with an argon current for three minutes before the autoclave plug was closed.

The accelerated thermodegradation at the elevated temperature was considered according to Van’t Hoff (for every 10 degree increase in reaction temperature, the reaction rate increases 2–4 times). During the tests, the temperature increased by 60 degrees: (140 °C–80 °C = 60 °C).

The fundamental basis of this approach is Van’t Hoff’s rule, which is mathematically expressed as the Equation:

ν_(T2)_ = ν_(T1)_·γ^(T2−T1)/10^ (2)

where ν(T2) and ν(T1) are reaction rates, respectively, at temperatures T2 and T1 (T2 > T1); γ is the temperature coefficient of the reaction rate.

The physical meaning of the coefficient γ is how many times the reaction rate changes for every 10 °C change in temperature. For many reactions, this coefficient lies in the range from 2 to 4. In the present work, the value of γ was taken to be 2.5. Thus, when increasing the temperature from 80 °C to 140 °C, the reaction rate increases 244 times, and 30 h of holding at 140 °C corresponds to approximately 10 months of holding at 80 °C.

Table 6 summarizes the conversion time parameters.

### 4.4. Filtration Studies

**Figure 6:**
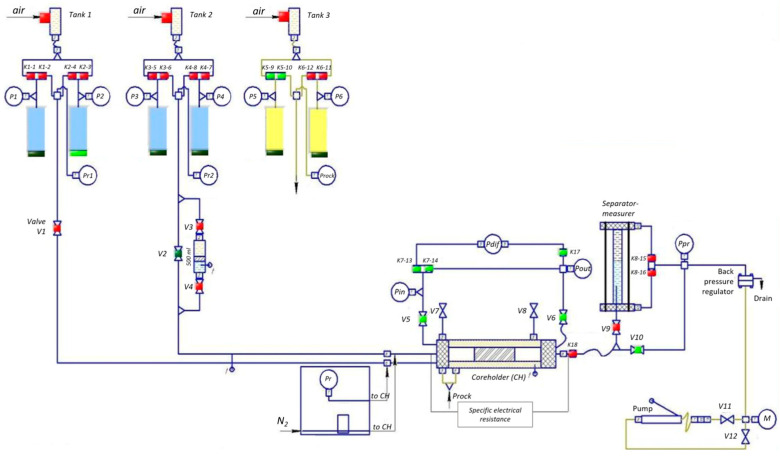
Schematic diagram of experimental unit SMP-FES-2R.

Preparation of core material and filtration studies were carried out according to the requirements of [31] on the core filtration analysis unit SMP-FES-2R (Kortekh, Mytishchi, Russia). The schematic diagram of the experimental unit SMP-FES-2R is presented in Figure 6, and technical characteristics are described in Table 7.

**Figure 7:**
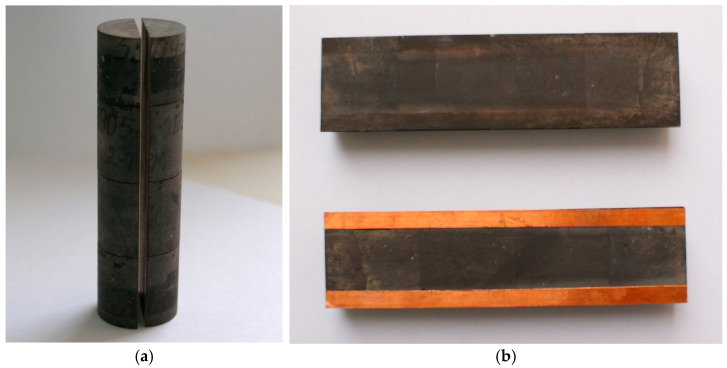

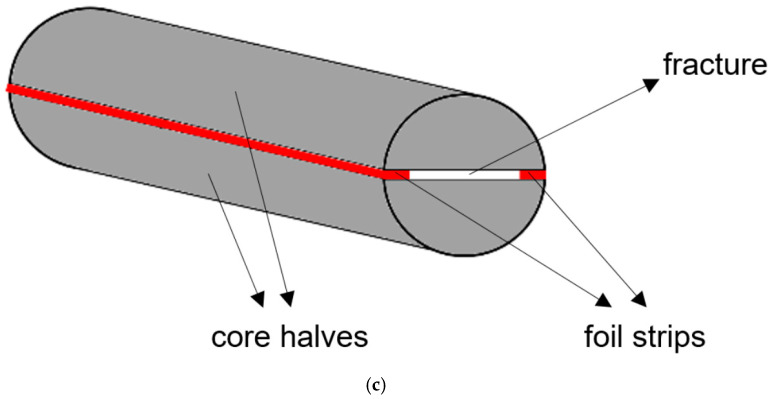
Photo of the ideal fracture model. (**a**) photo of the sawn core; (**b**) photo of the sawn halves of the core with glued foil strips; (**c**) scheme of an ideal fracture [32].

When creating a model of an ideal fracture (slot model—Figure 7), natural core samples were used, which made it possible to satisfactorily reproduce the conditions of natural wettability.

Later, cylindrical core samples were glued together to make a composite model with a length of at least 12.8 cm. The obtained composite porous media model was sawed lengthwise, and then the halves were matched so that the slotted model had a cylindrical shape. After polishing the contact surfaces, strips of copper foil of the appropriate thickness were glued to one of the halves (to form a given slot opening). The parameters of the created ideal fracture model were as follows (cm): length 12.8; width 3.0; nominal gap (slot opening) 0.05 cm. The orientation was horizontal. The surface of the slot model was carefully treated before the experiment by first cleaning off the dirt and then washing it with water and an alcohol solution.

For the second experiment, a composite porous model consisting of 3 core samples was prepared. The total length of composite model is 9 cm, its water permeability is 332.3 mD.

The following filtration testing procedure was used. The reservoir model (slot or composite) was placed in the core holder of the SMP FES-2R unit and created thermobaric conditions of the studied formation (80 °C). Then, the formation water model was filtered until the pressure drop stabilized (but not by less than 10 pore volumes).

After that, the tested composition was injected into the porous medium model in the volume of 1 pore volume for the slot model (for complete replacement of liquid in the slot space) and 0.5 pore volume for the porous composite model. After that, the system was technologically sedimented in statics for at least 20 h. Then, water injection was resumed with a constant flow rate (0.1 or 1.0 cm^3^/min) until stabilization of the pressure gradient was achieved, and permeability and maximum pressure drop were determined at each stage.

As a result, the residual resistance factor (*RRF*) was calculated—the ratio of pressure drop across the water after injection of the composition to the pressure drop before gel injection:

(3) $$RRF=\frac{dP_{i}}{dP_{1}}$$

where *dP_i_*—pressure drop after gel injection, MPa; *dP*_1_—pressure drop before gel injection, MPa.

## Figures and Tables

**Table 1:** Effect of nanosilica additives on gelation time and USS.

Additive Content, % wt.	Gelation Time, h	USS (Average Value from 3 Measurements), Pa
Hydrophobic RX380		
0.1	7	117
0.2	5.7	133
0.3	5	129
0.4	4.5	140
0.5	4.2	146
Hydrophilic HCSIL200		
0.1	7	140
0.2	7	198
0.3	7	258
0.4	7	290
0.5	7	339

**Table 2:** Results of oscillation experiments.

Composition	Elastic Modulus G’, Pa/St. dev., Pa	Viscous Modulus G’’, Pa/St. dev., Pa	Complex Modulus G*, Pa/St. dev., Pa	Complex Viscosity µ*, Pa⋅s/St. dev., Pa⋅s	Crossover Point, Pa/St. dev., Pa	LVR, Pa/St. dev., Pa
Base gel	36.1/0.9	20.9/1.0	41.7/1.3	6.6/0.2	50.5/1.6	28.7/2.3
Gel + 0.4% HCSIL200	57.3/5.5	29.6/2.7	64.5/6.2	10.3/1.0	67.4/12.4	37.0/4.0
Gel + 0.4% HCSIL300	49.7/5.3	25.7/1.8	56.0/5.6	8.9/0.9	82.9/22.3	35.8/6.7
Gel + 0.4% RX380	37.2/4.4	18.9/2.8	41.8/5.2	6.6/0.8	50.7/7.1	31.0/6.1
Gel + 0.4% BS-50	36.8/4.3	18.8/1.9	41.3/4.7	6.6/0.7	46.5/1.1	29.0/0.2
Gel + 0.4% BS-120 NU	45.7/0.4	23.7/1.8	51.5/1.9	8.2/0.3	59.6/4.3	34.5/2.9
Gel + 0.4% BS-120 U	39.2/5.5	20.9/2.3	44.4/6.0	7.1/1.0	44.0/10.5	29.6/6.4

**Table 3:** Parameters of porous media and results of filtration experiments.

Test №	Model	Water Permeability, mD		Pressure Difference, MPa		Flow Rate, cm^3^/min	Vpore, u. f.	Maximum Pressure Drop, MPa	*RRF*, u. f.
		Before	After	Before	After				
1	Slot model (opening 0.05 cm)	not measured	not measured	0.0027	0.6112	0.1	1.0	0.97	226.4
				0.0082	0.7507	1.0		1.5	91.6
2	Composite porous model	332.3	30.1	0.2729	3.000	0.1	0.5	9	11.0

**Table 4:** USS of hydrogels before and after thermal degradation.

Hydrogels	USS of the Sample Under Thermal Degradation, Pa		Exposure Time, T = 140 °C, h	Calculated Time, T = 80 °C, Month	Photo After Thermal Degradation
	Before	After			
Base gel	104	92.5	15	5	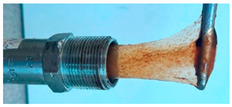
		72.0	26	9	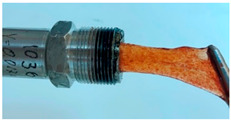
		69.0	57	19	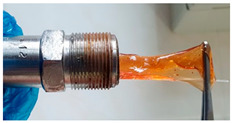
Base gel + 0.4% HCSIL200	243	208	15	5	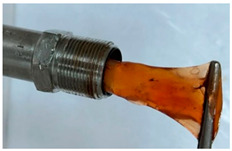
		180	26	9	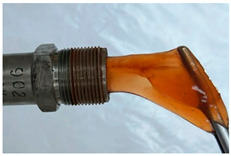
		116	57	19	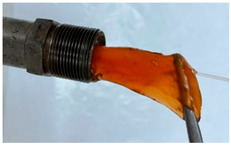
		Destructed	68	23	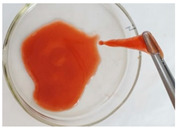
Base gel + 0.4% BS-120 NU	116	73	21	7	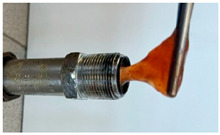
		69	29	10	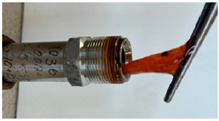
		66	57	19	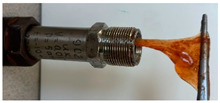

**Table 5:** Characteristics of nanodispersed fillers.

Nanoadditive	Specific Surface, m^2^/g	Calculated Mean Diameter, nm
HCSIL200	202	13.5
HCSIL300	301	9.1
RX380	380	7.2
BS-50	39	69.9
BS-120 U	108	25.3
BS-120 NU	132	20.7

**Table 6:** Recalculation parameters for thermal acceleration.

Speed Increase Factor	Acceleration	Exposure Time at Temperature 140 °C, h			
2.06	64	33.0	66	99.0	135.0
2.56	244	8.8	17.7	26.6	35.4
3.06	729	2.9	5.9	8.9	11.8
Recalculated time value corresponding to real reservoir conditions		3 months	6 months	9 months	12 months

**Table 7:** The technical characteristics of the SMP-FES-2R unit.

№	Characteristic	Value
1	Linear length of the core model, mm	100–300
2	Core temperature control range, °C	+25–+150
3	Maximum rock pressure, MPa	70
4	Maximum reservoir pressure, MPa	55

## Data Availability

The data presented in this study are available on request from the corresponding author.
